# PyGlaucoMetrics: A Stacked Weight-Based Machine Learning Approach for Glaucoma Detection Using Visual Field Data

**DOI:** 10.3390/medicina61030541

**Published:** 2025-03-20

**Authors:** Mousa Moradi, Saber Kazeminasab Hashemabad, Daniel M. Vu, Allison R. Soneru, Asahi Fujita, Mengyu Wang, Tobias Elze, Mohammad Eslami, Nazlee Zebardast

**Affiliations:** 1 Harvard Ophthalmology AI Lab, Schepens Eye Research Institute of Massachusetts Eye and Ear, Harvard Medical School, Boston, MA 02114, USA; mmoradi2@meei.harvard.edu (M.M.); skazeminasabhashemabad@meei.harvard.edu (S.K.H.); mengyu_wang@meei.harvard.edu (M.W.); tobias_elze@meei.harvard.edu (T.E.); mohammad_eslami@meei.harvard.edu (M.E.); 2 Massachusetts Eye and Ear, Harvard Medical School, Boston, MA 02114, USA; daniel_vu@meei.harvard.edu (D.M.V.); allison_soneru@meei.harvard.edu (A.R.S.); afujita@meei.harvard.edu (A.F.)

**Keywords:** glaucoma, classification, Humphrey field analyzer, machine learning, MLP

## Abstract

*Background and Objectives*: Glaucoma (GL) classification is crucial for early diagnosis and treatment, yet relying solely on stand-alone models or International Classification of Diseases (ICD) codes is insufficient due to limited predictive power and inconsistencies in clinical labeling. This study aims to improve GL classification using stacked weight-based machine learning models. *Materials and Methods*: We analyzed a subset of 33,636 participants (58% female) with 340,444 visual fields (VFs) from the Mass Eye and Ear (MEE) dataset. Five clinically relevant GL detection models (LoGTS, UKGTS, Kang, HAP2_part1, and Foster) were selected to serve as base models. Two multi-layer perceptron (MLP) models were trained using 52 total deviation (TD) and pattern deviation (PD) values from Humphrey field analyzer (HFA) 24-2 VF tests, along with four clinical variables (age, gender, follow-up time, and race) to extract model weights. These weights were then utilized to train three meta-learners, including logistic regression (LR), extreme gradient boosting (XGB), and MLP, to classify cases as GL or non-GL. *Results*: The MLP meta-learner achieved the highest performance, with an accuracy of 96.43%, an F-score of 96.01%, and an AUC of 97.96%, while also demonstrating the lowest prediction uncertainty (0.08 ± 0.13). XGB followed with 92.86% accuracy, a 92.31% F-score, and a 96.10% AUC. LR had the lowest performance, with 89.29% accuracy, an 86.96% F-score, and a 94.81% AUC, as well as the highest uncertainty (0.58 ± 0.07). Permutation importance analysis revealed that the superior temporal sector was the most influential VF feature, with importance scores of 0.08 in Kang’s and 0.04 in HAP2_part1 models. Among clinical variables, age was the strongest contributor (score = 0.3). *Conclusions*: The meta-learner outperformed stand-alone models in GL classification, achieving an accuracy improvement of 8.92% over the best-performing stand-alone model (LoGTS with 87.51%), offering a valuable tool for automated glaucoma detection.

## 1. Introduction

Glaucoma (GL) is a major cause of irreversible blindness worldwide [1]. The disease is characterized by progressive retinal ganglion cell (RGC) loss, leading to permanent visual field (VF) damage [2,3]. Standard automated perimetry (SAP) is widely regarded as the gold standard for tracking visual function in GL patients [4]. However, SAP has inherent limitations, including subjectivity, test-retest variability, and confounding effects from age-related visual changes, all of which can reduce its effectiveness in detecting GL and accurately assessing functional impairment [3,5,6,7]. Establishing a robust framework for visualizing, statistically analyzing, and clinically interpreting VF loss is essential for effective GL management. Additionally, there is critical research need for precise methodologies to identify and quantify vision loss caused by GL. Current methods of relying on International Classification of Diseases (ICD) diagnosis codes and manual review of VF records are inconsistent and labor intensive [8,9].

Machine learning (ML) has emerged as a powerful tool in ophthalmology, improving disease classification, progression prediction, and treatment planning [10,11,12,13,14,15]. In VF analysis, ML models can detect glaucomatous patterns more effectively than conventional statistical approaches. Artes et al. (2005) employed a pointwise linear regression model to evaluate VF progression by comparing total deviation (TD) and pattern deviation (PD) analyses in a prospective longitudinal study involving GL patients and healthy controls [16]. Their findings indicated that PD might underestimate VF progression in GL, especially in the absence of clinical signs of worsening media opacity. Later on, Sabharwal et al. (2023) demonstrated that deep learning models can reliably predict VF progression with an area under the curve (AUC) of 0.94, incorporating both trend- and event-based methods [17].

Five clinical criteria [18] have been widely used in GL research and clinical practice to define VF defects, including LoGTS [19] (Low-Pressure Glaucoma Treatment Study) by Krupin et al. (2005), UKGTS [20,21] (United Kingdom Glaucoma Treatment Study) by Garway-Heath et al. (2013 and 2015), Kang’s method [22] by Kang et al. (2015), HAP2 [23,24] (Humphrey Automated Perimetry Part 2) by Perkins and Chang et al. (1994 and 2016), and Foster [25] criterion by Foster et al. (2002). Despite their clinical value, these criteria vary in predictive performance across different datasets. A key challenge in GL classification is integrating multiple diagnostic criteria into a unified and reliable model.

Previous research has led to the development of VF analysis tools in R and Python. Marín-Franch et al. (2013) created an R-based VF packages for analyzing and visualizing VFs, but its effectiveness varied across datasets, making standardization challenging [26]. Later, Elze et al. (2015) introduced the “vfprogression” package in R, designed specifically for VF progression analysis [27], yet it lacked comprehensive diagnostic capabilities. While these tools are valuable for tracking changes over time, there is still no toolbox that provides an ML-based framework for GL classification within a unified approach.

To address these gaps, we propose a stacked weight-based meta-learning framework to improve GL detection based on VF data. In this approach, we first train multi-layer perceptron (MLP) models using TD and PD values from Humphrey field analyzer (HFA) 24-2 VF tests to extract model weights. These extracted weights capture meaningful features from VF data, incorporating multiple clinical criteria. We then use these weights as inputs for three meta-learners—namely, logistic regression (LR) [28], extreme gradient boosting (XGB) [29,30], and MLP [31]—to classify VFs as GL or non-GL. Our primary contribution in this study is the development of a novel stacked-weight meta-learning approach, which integrates the predictive strengths of multiple ML-based models to improve GL detection, with a particular focus on diagnosis rather than disease monitoring. Unlike traditional single-model approaches, our framework leverages stacked model weights to enhance predictive accuracy and robustness. By testing different meta-learning models, we assess their ability to capture VF data complexity and optimize GL detection performance. Additionally, our secondary goal is to provide an open-source toolbox, PyGlaucoMetrics, which enables all analyses within a user-friendly environment. By integrating multiple clinical criteria into a structured ML pipeline, our approach advances the field of automated GL detection, offering a more reliable, accessible, and scalable method for VF-based diagnosis.

## 2. Materials and Methods

### 2.1. Dataset and Clinical Labeling

Standard automated perimetry tests from study participants were obtained using the HFA II (Carl Zeiss Meditec, Inc., Dublin, CA, USA) at Mass Eye and Ear (MEE) of Mass General Brigham (MGB). The study received approval from the Institutional Review Board at MGB, and all procedures complied with the principles outlined in the Declaration of Helsinki for research involving human participants. Since this study involves the use of secondary data, informed consent is not necessary, and our IRB has granted a waiver for this requirement. Pointwise sensitivities were extracted from HFA 24-2 VF tests. The HFA 24-2 test pattern was selected for training our model because it is widely regarded as the standard method in glaucoma diagnosis and is commonly used in clinical practice [32]. TD and PD values were computed for all 52 test locations, excluding the two blind spots, using the PyVisualFields v. 1.0.4 [33]. VF data with a false positive rate (FPR) exceeding 33% were excluded [34,35]. Only patients with at least two VF tests conducted on different dates were considered, and a minimum follow-up period of six months was required for inclusion [36]. However, despite the inclusion of multiple tests per patient, this study was cross-sectional rather than longitudinal, meaning it did not analyze disease progression over time. These data were utilized for both training and validation purposes. The final dataset comprised 33,636 patients with 340,444 VF tests, and model performance was assessed through a clinical review. A random subset of 200 patients was selected for clinical review. Two fellowship-trained glaucoma specialists independently examined the corresponding clinical records to confirm the diagnosis of GL. Only the patients for whom both reviewers agreed on the diagnosis (GL or Non-GL) were included, resulting in 160 VFs from 160 patients, reflecting an 80% inter-rater agreement, which is consistent with prior reports in glaucoma diagnosis [37]. This approach aimed to minimize the inherent subjectivity in clinical diagnosis and ensure high confidence in the clinical labels used for model training. These data were then used for the training and testing of three meta-learner models. The dataset was split into 82.5% for training (N = 132) and 17.5% for testing (N = 28).

### 2.2. Stand-Alone Model Development

We developed five independent models from scratch to classify glaucomatous VFs. These models are HAP2_part1 (HAP2_p1), Foster, UKGTS, Kang, and LoGTS. Supplementary Table S1 shows the full criteria used in each model to classify glaucomatous VFs. The input data for each model consist of either 52 TD or PD values. All scripts for these models are publicly available on the first author’s GitHub page as v.1.0. The models were implemented in Python v. 3.8.19. In cases where TD or PD values were missing for some examinations, they were recovered using sensitivity data from the PyVisualFields v. 1.0.4 library [33]. Other essential libraries, such as PyQt5 v. 5.15.10 (for GUI development) and rpy2 v. 3.4.5 (the wrapper library), were installed via pip. Data preprocessing and analysis were carried out with Pandas v. 1.2.4 and NumPy v. 1.24.4, while visualization was performed using Matplotlib v. 3.7.3 and Seaborn v. 0.13.0.

### 2.3. Data Preprocessing and Training Protocol

Missing values were addressed using imputation by filling them with the median value of each column, while categorical variables such as race and gender were converted into numerical labels to ensure compatibility with the model. The base models consisted of two TD-based classifiers (HAP2_p1, Foster) and three PD-based classifiers (UKGTS, LoGTS, Kangs), all adjusted for clinical data (age, race, gender, and follow-up time). Given the moderate to strong correlation between the outputs of these five models (Supplementary Figure S2), a single MLP was trained as the base model [38]. For feature extraction, two MLPs were trained using a combination of 52 TD or PD features and 4 clinical variables (HAP2_p1 and Foster were trained on PD data, and UKGTS, LoGTS, and Kang on TD data). Hyperparameters were optimized using the “GridSearchCV” class from the scikit-learn library with 5-fold cross-validation. “GridSearchCV” searched for the best combination of hyperparameters, including activation functions [‘softmax’, ‘ReLU’, ‘sigmoid’], learning rates [1 × 10^−2^, 1 × 10^−3^, 1 × 10^−5^], and optimizers [‘SGD’, ‘RMSprop’, ‘Adam’]. The optimal configuration was found to be an activation function of ‘sigmoid’, a learning rate of 1 × 10^−3^, and an optimizer of ‘Adam’. The number of epochs was set to 25, with early stopping enabled and a patience of 3. To enhance the input features for the models, clinical data (age, race, follow-up time, and gender) were incorporated alongside the TD and PD features for each of the five stand-alone models. This modification ensured the model accounted for the influence of demographic and clinical factors on the prediction, resulting in a more robust, context-aware model that considered both medical and patient-specific variables.

### 2.4. The Proposed Meta-Learners

**Figure 1:**
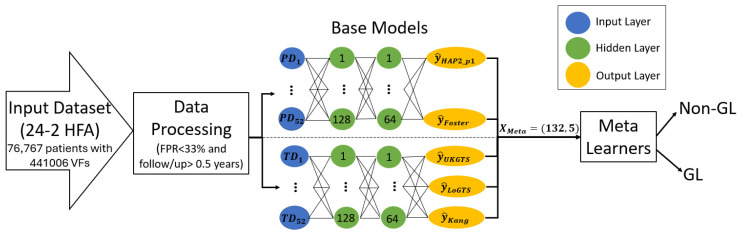
General block diagram of the proposed stack weight classification model. Besides TD and PD values, clinical data including age, race, gender, and follow-up time were included during weight extraction in each base learner. Follow/up = Follow-up time.

After extracting weights using the output layer of the base models, the three meta-learners (LR, XGB, and MLP) were trained to combine the output features. The selection of these models was driven by their ability to efficiently handle structured tabular data [39,40,41,42], as each VF sample was represented as a 132-dimensional feature vector rather than an image or sequential data [43,44]. The selection of these models ensured a balance between model complexity, interpretability, and predictive power. The MLP model had 3 layers, with 128 neurons in the first layer, 64 neurons in the second, and a sigmoid activation function in the final layer. The model used batch normalization and ‘ReLU’ activation between the hidden layers, along with a dropout rate of 0.08 to avoid overfitting. The hyperparameters include the Adam optimizer, a learning rate of 0.0031, and a weight decay of 1 × 10^−3^, using a batch size of 32. For the other meta-learners, the LR model was trained using the ‘LogisticRegression’ class from scikit-learn with L2 regularization, a solver of ‘lbfgs’, and no penalty term (C = 1.0). The XGB model was trained using the ‘XGBClassifier’ from the XGBoost library with a learning rate of 0.3, a maximum depth of 6, and a boosting type of ‘gbtree’. All models were trained on the meta-features extracted from the base models’ outputs, and their hyperparameters were optimized for best performance. All models were trained for 25 epochs. Figure 1 shows the block diagram of the proposed model.

### 2.5. Statistical Analysis

The Wilcoxon signed-rank test, from the Python “stat” library, was applied for pairwise model comparisons. Tests with a significance level of <0.05 were considered statistically significant. To remove outliers from the analysis, the IQR for each model’s probabilities was computed. To assess uncertainty in the predictions of the meta-learner models (logistic regression, XGB, and MLP), the entropy for each model was calculated using the following formula:

(1) $$H\left(p\right)=-(p\log\left(p\right)+(1-p)\log\left(1-p)\right)$$

where *p* is the predicted probability for the GL class and 1 − *p* is the predicted probability of the non-GL class.

## 3. Results

### 3.1. Patients Characteristics

Table 1 presents the characteristics of the 33,636 study participants (mean age 61.86 ± 14.40, 58% female) with 340,444 VFs. The majority of participants identified as White (70.18%, 309,516), followed by Black/African Americans (11.70%, 51,579), Asians (6.02%, 26,563), American Indian/Alaska Native (3.98%, 17,564), and others (7.59%, 33,473). The median follow-up time was 1.25 years, with an interquartile range (IQR) of [0, 4.93] years. On average, each eye had 2.95 visits (SD = 3.35). At baseline, the mean MD was −4.48 dB (SD = 6.49). Patients were categorized into three groups based on their MD values [45] into mild MD (MD > −4.20) with a mean MD of −1.13 dB (SD = 1.73), moderate MD (−8.17 < MD ≤ −4.20) with mean MD of −5.83 dB (SD = 1.12), and severe MD (MD ≤ −8.17) with mean MD of −16.34 dB (SD = 6.70).

### 3.2. Permutation Importance Analysis

**Figure 2:**
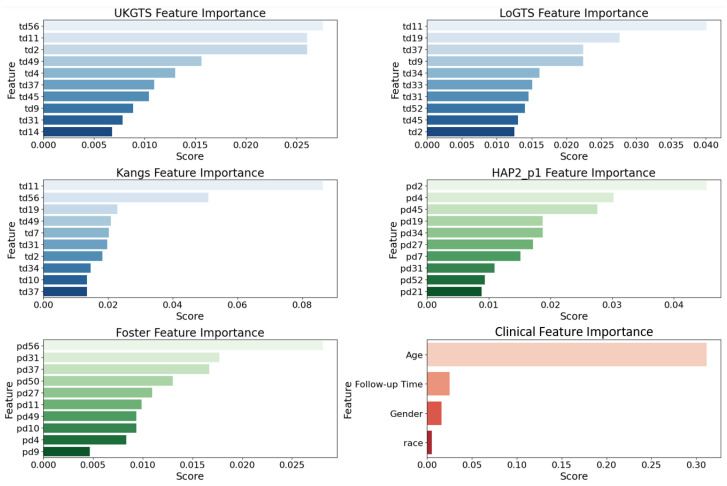
The top 10 MLP-derived feature importance scores. Important features were based on the weights of each stand-alone model extracted by MLP. TD = total deviation, and PD = pattern deviation values.

To assess the relative contribution of different features in our base models, we utilized permutation importance [46] with feature values randomly shuffled. This method ensures a more reliable ranking by mitigating biases from collinear features (e.g., age). The top 10 influential features (Figure 2) were largely associated with the superior temporal (ST) and inferior nasal (IN) sectors. In the UKGTS model, key features included td11 (ST, 0.026), td2 (ST, 0.023), and td37 (IN, 0.018), while in LoGTS, the most important were td11 (ST, 0.039), td19 (ST, 0.034), and td34 (IN, 0.028). The Kangs model showed the highest permutation scores, with td11 (ST, 0.080), td19 (ST, 0.065), and td34 (IN, 0.050) among the top predictors. Conversely, in the HAP2_p1 and Foster models, pd2 (ST, 0.041), pd4 (ST, 0.038), and pd34 (IN, 0.032) exhibited the highest importance scores. Furthermore, pd31 (IN, 0.027), pd52 (IN, 0.025), and pd21 (ST, 0.022) were identified as key contributors in HAP2_p1, whereas pd56 (IN, 0.026), pd31 (IN, 0.023), and pd37 (ST, 0.021) ranked as the most significant features in Foster. As expected, age was the most influential clinical factor in all models (permutation score ~0.30), followed by follow-up time, while gender and race had a minimal predictive impact. The list of all features is provided in Supplementary Figure S1.

### 3.3. Model Execution Results

Table 2 shows the performance metrics of the developed meta-learners, and stand-alone models demonstrate that the MLP achieved the highest accuracy (96.43%), precision (92.32%), sensitivity (100%), and F-score (96.01%) among all models. The XGB model follows with an accuracy of 92.86%, precision of 85.71%, and an F-score of 92.31%. LR attained an accuracy of 89.29%, precision of 90.91%, and a sensitivity of 83.33%. Among the stand-alone models, LoGTS exhibited the highest accuracy (87.51%) and an F-score of 83.33%. UKGTS and Kang demonstrated comparable performances, with accuracy values of 84.40% and 84.41%, respectively. The Foster model achieved the lowest accuracy (65.65%) and precision (52.22%) but retained a high sensitivity of 95.03%.

**Figure 3:**
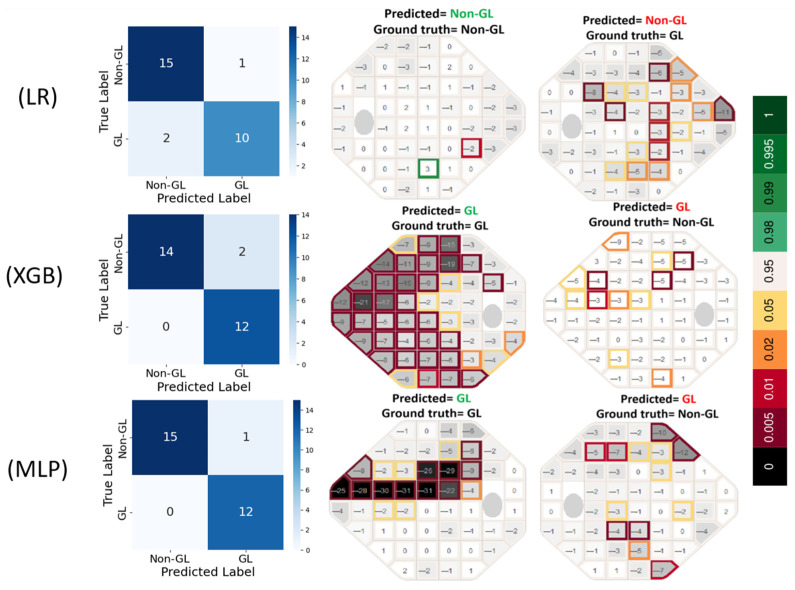
Confusion matrices and prediction results for three meta-learners. The labels ‘LR’, ‘XGB’, and ‘MLP’ refer to the respective three meta-learner classifiers used in this study. The green and red colors in prediction plots indicate correct and incorrect predicted labels, respectively. The color bar represents the statistical probability that a specific point on the VF is considered as normal. LR = logistic regression, XGB = extreme gradient boosting, and MLP = multi-layer perceptron.

The confusion matrices in Figure 3 reveal that LR produced one false positive (FP) and two false negative (FN) errors, suggesting a tendency to under-detect glaucomatous cases. In contrast, XGB completely eliminated FNs but resulted in two FP errors, indicating a slight tendency to overpredict GL cases. MLP exhibited the most accurate classification performance, with only one FP and zero FN errors, highlighting its effectiveness in capturing glaucomatous patterns without misclassifying true GL cases. The prediction VF plots are further supporting these results.

**Figure 4:**
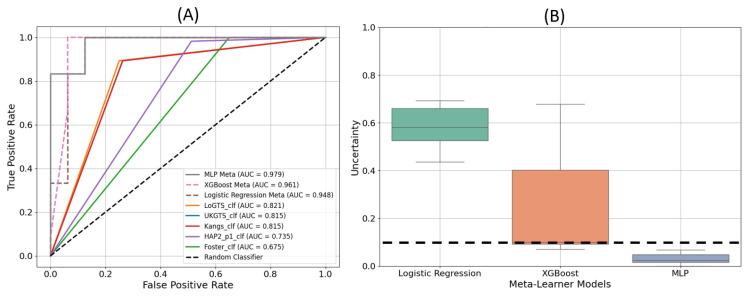
ROC curves for stand-alone and meta-learner models (**A**), uncertainty in predictions for each meta-learner models (**B**). The dashed line indicates the uncertainty threshold of 10%.

The receiver operating characteristic (ROC) curves in Figure 4A demonstrate the performance of each model in distinguishing glaucomatous from non-glaucomatous VFs. The MLP meta-learner achieved the highest AUC value of 0.979, followed by XGB with an AUC of 0.961, and LR with an AUC of 0.948. Among the stand-alone models, LoGTS exhibited the highest AUC (0.821), whereas the Foster model had the lowest (0.675). Figure 4B displays the uncertainty in predictions for the three meta-learner models. The logistic regression model exhibited the highest level of uncertainty, while MLP demonstrated the lowest, remaining well below the uncertainty threshold of 10%.

As shown in Table 3, the proposed MLP meta-learner in this study outperformed similar reported models in the literature. Specifically, the MLP achieved an accuracy of 96.43%, a precision of 92.32%, and a sensitivity of 100%, with an AUC of 97.96%. Comparatively, Wu et al. (2021) [47] reported an accuracy of 87.1% and an AUC of 94% using a decision tree model, while Masumoto et al. (2018) [48] achieved a sensitivity of 81.3% and an AUC of 87.2% using a deep learning model. The performance of the proposed MLP model demonstrates a substantial improvement over the prior method.

## 4. Discussion

In this study, we introduce PyGlaucoMetrics as a stacked weight-based meta-learning approach, which integrates the predictive strengths of multiple ML-based models to improve GL detection. Unlike prior studies that rely on single-model approaches, such as Marín-Franch et al.’s R-based visualFields package [26] and Elze et al.’s vfprogression package [27], PyGlaucoMetrics provides a meta-learning framework, integrating predictions from multiple established models to improve classification accuracy and robustness. By leveraging a meta-learner trained on model outputs rather than simple majority voting, PyGlaucoMetrics enhances standardization, interpretability, and reproducibility in GL detection and severity assessment.

Our meta-learning approach integrates outputs from five well-established VF-based classifiers (HAP2, UKGTS, LoGTS, Kang’s method, and Foster) and refines predictions through a secondary learning stage. Three machine learning models including LR, XGB, and MLP were developed as meta-learners, trained on the outputs of the base classifiers. Among these, MLP outperformed all other models, achieving an accuracy of 96.43%, precision of 92.32%, and AUC of 97.96%, demonstrating superior ability to distinguish GL from non-GL VFs. In comparison, XGB and LR achieved AUCs of 96.1% and 94.8%, respectively, while LoGTS achieved an AUC of 82.1%, the highest among stand-alone classifiers. The improved classification performance of the meta-learners, particularly MLP, highlights the advantage of aggregating information from multiple VF models rather than relying on a single classification criterion.

Performance validation using an independent test set further confirmed the clinical reliability of PyGlaucoMetrics. The MLP meta-learner demonstrated the lowest uncertainty in predictions, as indicated in Figure 4B, while LR exhibited higher uncertainty levels, suggesting a greater sensitivity of MLP to robust VF patterns. Additionally, feature importance analysis (Figure 2) identified the superior temporal (ST) and inferior nasal (IN) sectors as key regions in glaucoma classification, with td11 (ST, 0.080) and td34 (IN, 0.050) ranking highest in the Kangs model, consistent with the UKGTS and LoGTS models, which also prioritized td11 (ST). These findings align with clinical knowledge, as the ST and IN regions are often among the first to show glaucomatous damage. The ST area is vulnerable due to its proximity to the retinal nerve fiber layer, which is highly susceptible to early damage in glaucoma [49,50,51]. Also in this study, we included all major glaucoma subtypes, including primary open-angle glaucoma (POAG), primary angle-closure glaucoma (PACG), pseudoexfoliation glaucoma (PXG), normal-tension glaucoma (NTG), hypertensive glaucoma (HTG), traumatic glaucoma (TG), and neovascular glaucoma (NVG). While the progression patterns may differ slightly across these subtypes, the ST and IN sectors consistently showed high importance in classification. This suggests that these regions remain critical markers of glaucomatous damage, regardless of subtype, and reflect common patterns of functional loss across different forms of the disease [52,53]. In contrast, pd2 (ST, 0.041) and pd34 (IN, 0.032) contributed more to the HAP2_p1 and Foster models. Age was the most influential clinical variable across all models (~0.30), while gender and race had minimal impact. These findings reinforce the role of ST and IN sectors in GL progression and suggest that integrating both TD- and PD-derived features enhances predictive performance.

The clinical applicability of PyGlaucoMetrics was assessed using a cohort of 160 patients, where MLP outperformed traditional stand-alone classifiers, achieving an AUC of 96% in distinguishing GL vs. non-GL eyes. Compared to existing methods, PyGlaucoMetrics demonstrated a notable improvement, surpassing the C5 decision tree model by Wu et al. (AUC = 94%) and the deep learning model by Masumoto et al. (AUC = 87.2%). These results highlight the advantage of the meta-learning framework in reducing bias and improving model generalizability across different datasets.

Despite its strong performance, several limitations of PyGlaucoMetrics should be acknowledged. One major challenge in VF-based classification is distinguishing glaucoma from other optic neuropathies, such as early chiasmal compression, past anterior ischemic optic neuropathy (AION) [54], or branch retinal artery occlusion (BRAO) [55]. These conditions can present with overlapping visual field defects, making differential diagnosis an important consideration. However, training a model to differentiate these conditions is challenging due to their relative rarity compared to POAG. ML models require large, well-balanced datasets, and the prevalence of these conditions is significantly lower than POAG, which makes obtaining sufficient training data difficult. Prior studies have also highlighted the limitations of AI-based models in handling rare ophthalmic diseases due to dataset imbalances [56]. Furthermore, dual diagnosis scenarios, where glaucoma coexists with other conditions, present an even greater challenge due to overlapping structural and functional deficits. While refining protocols for differential diagnosis is a valuable goal, addressing dual diagnosis cases in an AI model would require significantly larger and more diverse datasets, which are currently not widely available. Future work should explore how the model performs in patients with coexisting ocular or neurological conditions.

Additionally, the lack of a universally accepted standard for glaucoma diagnosis indeed complicates the benchmarking of model performance. To address this, we ensured that the clinical labels in our study were validated by two fellowship-trained glaucoma specialists, achieving a high inter-rater agreement of 80%, which is consistent with typical findings in clinical settings [37]. However, we recognize that even with expert consensus, subjectivity may still influence the diagnosis. In future work, we aim to refine the diagnostic process by incorporating additional data sources, such as longitudinal VF trends and multimodal imaging (e.g., optical coherence tomography (OCT)), to improve diagnostic accuracy and standardization. Furthermore, exploring consensus-based guidelines or integrating larger datasets across multiple clinical centers may help mitigate the current variability and improve the reliability of the model across diverse patient populations and clinical settings. Although this study primarily focused on 24-2 VF data, our proposed model is also compatible with the 10-2 test pattern. Supplementary Figure S3 shows this compatibility. The 10-2 test is particularly valuable for detecting macular defects, which play a crucial role in the early stages of glaucoma, whereas the 24-2 pattern is more effective for identifying glaucomatous damage across a broader spectrum [32], which was the primary focus of our study. Incorporating the 30-2 and Octopus perimeters requires additional considerations due to variations in test locations, grid spacing, and calibration methods [57,58]. These structural differences necessitate further modifications to the model. However, we plan to enhance the model’s functionality in the next version to support additional data types, including the 30-2. Future work will address this limitation by incorporating cross-validation with these alternative testing protocols, aiming to enhance the model’s generalizability and robustness across diverse clinical settings.

Although this study focuses on VF-based classification, OCT has been widely used in glaucoma assessment and could complement VF analysis [59,60]. Integrating OCT-derived structural data, such as retinal nerve fiber layer thickness, could enhance the model’s diagnostic accuracy and robustness. Future research should investigate whether a combined OCT-VF model could outperform current VF-based classifiers in both diagnosis and disease monitoring [59]. While no widely accepted OCT-VF fusion algorithm for GL detection currently exists, recent studies suggest that deep learning approaches may facilitate such integration for disease progression [61]. Future research should also assess whether combining VF-based functional deficits with OCT-derived structural changes could lead to earlier and more accurate glaucoma detection. Further exploration of deep learning models for time-series VF analysis could enhance PyGlaucoMetrics’ ability to predict glaucoma progression, extending its utility beyond cross-sectional classification. While PyGlaucoMetrics shows promise in automated GL detection, it is currently more suited for research than clinical use. PyGlaucoMetrics has been tested on our internal clinical data, with promising results. Our next steps include evaluating PyGlaucoMetrics on external clinical datasets to ensure its generalizability across different patient populations and clinical environments. Additionally, incorporating feedback from ophthalmologists will be key to assessing the model’s usability in real-world clinical workflows.

## 5. Conclusions

PyGlaucoMetrics, as a stacked weight-based meta-learning approach, significantly improves GL classification by integrating outputs from multiple VF-based models. Among the meta-learners, MLP demonstrated superior performance with minimal misclassification errors and the most distinct probability distribution for GL detection. The findings suggest that utilizing meta-learning enhances classification robustness and generalizability, offering a valuable tool for automated glaucoma detection.

## Figures and Tables

**Table 1:** Clinical characteristics of the subjects included in this study. Data are presented as mean (SD), median [IQR] or *n* (%). Other races include Hispanic, Hawaiian, and not reported races.

Characteristic	
Age at baseline, years, Mean (SD)	61.86 (17.40)
Gender, *n* (%)	
Female	19,528 (58.06%)
Male	14,109 (41.95%)
Race, *n* (%)	
White	309,516 (70.18%)
Black/African Americans	51,579 (11.70%)
Asians	26,563 (6.02%)
American Indian/Alaska Native	17,564 (3.98%)
Others	33,473 (7.59%)
Visual fields (total), *n*	340,439
Follow-up time, years, median [IQR]	2.49 [0.54, 6.22]
N of visits per eye, years, Mean (SD)	5.16 (3.35)
MD at baseline, dB, Mean (SD)	
Overall	−4.48 (6.49)
Mild (MD > −4.20)	−1.13 (1.73)
Moderate (−8.17 < MD <= −4.20)	−5.83 (1.12)
Severe (MD <= −8.17)	−16.34 (6.70)

**Table 2:** Performance metrics for the developed meta-learners and stand-alone models. LR = logistic regression, XGB = extreme gradient boosting, and MLP = multi-layer perceptron. All metrics are in %.

Classifier	Accuracy (%)	Precision (%)	Sensitivity (%)	F-Score (%)
MLP	96.43	92.32	100	96.01
XGB	92.86	85.71	100	92.31
LR	89.29	90.91	83.33	86.96
LoGTS	87.51	76.92	90.90	83.33
UKGTS	84.40	73.30	91.72	81.48
Kang	84.41	73.32	91.73	81.50
HAP2_p1	78.14	63.22	95	75.92
Foster	65.65	52.22	95.03	67.40

**Table 3:** Comparison of the proposed meta-learner against similar works in the literature.

Method, Year	Test Type	Accuracy (%)	Precision (%)	Sensitivity (%)	AUC (%)
MLP Meta-Learner (this study), 2025	24-2 VF	96.43	92.32	100	97.96
Wu et al. [47], C5 Decision Tree, 2021	30-2 VF	87.1	84.7	88.3	94
Masumoto et al. [48], Deep learning model, 2018	24-2 VF	NA	80.2	81.3	87.2

## Data Availability

The data can be obtained from the corresponding author upon request. The code for this article is available from the first author GitHub page at: https://github.com/Mousamoradi/PyGlaucoMetrics (accessed on 16 March 2025).

## Supplementary Materials

The following supporting information can be downloaded at: https://www.mdpi.com/article/10.3390/medicina61030541/s1, Figure S1: List of all important features extracted by MLP and their contribution to each stand-alone model; Figure S2: Correlation of predictions in each stand-alone model. Values > 0.5 indicate moderate to high correlation. Pearson correlation coefficient was used to calculate all values; Figure S3: Examples of HFA data inputs compatible by the proposed model. (A) data_vfpwgRetest24d2: Short-term retest static automated perimetry data. Collected from 30 glaucoma patients at the Queen Elizabeth Health Sciences Centre in Halifax, Nova Scotia, with 12 visual field tests over 12 weekly sessions [62]. (B) data_vfpwgSunyiu24d2: 24-2 static automated perimetry data from a patient with glaucoma. This dataset contains real patient data with age modified for anonymity [63]. (C) data_vfctrSunyiu24d2: A dataset of healthy eyes for 24-2 static automated perimetry, used to generate normative values (sunyiu_24d2 and related datasets). Courtesy of William H. Swanson and Mitch W. Dul [63]. (D) data_vfctrSunyiu10d2: A dataset of healthy eyes for 10-2 static automated perimetry, also provided by William H. Swanson [63]; Table S1: Criteria used in our package to evaluate glaucoma defects. HAP2 = Hodapp-Anderson-Parrish 2; UKGTS = United Kingdom Glaucoma Treatment Study; LoGTS = Low-pressure Glaucoma Treatment Study. PDP = Pattern deviation plot, MD = Mean Deviation, TDP = Total deviation plot, TD = Total deviation, GHT = Glaucoma Hemifield Test.
