# Supplementary material for: PyGlaucoMetrics: A Stacked Weight-Based Machine Learning Approach for Glaucoma Detection Using Visual Field Data
Source: Medicina (Kaunas). 2025 Mar 20;61(3):541. doi: 10.3390/medicina61030541 (PMC11944261; doi:10.3390/medicina61030541)
Supplement: Supplementary file 1 [file medicina-61-00541-s001.zip › medicina-3509821-supplementary.pdf]

PyGlaucoMetrics: A Stacked Weight Based Machine Learning Approach for Glaucoma Detection Using Visual Field Data

Mousa Moradi [1], Saber Kazeminasab Hashemabad [1], Daniel M Vu [2], Allison R Soneru [2], Asahi Fujita [2], Mengyu Wang [1], Tobias Elze [1], Mohammad Eslami [1,*] and Nazlee Zebardast [2,*]

* co-senior authors, contributed equally to this work

[1] Harvard Ophthalmology AI Lab, Schepens Eye Research Institute of Massachusetts Eye and Ear, Harvard Medical School, Boston, MA, United States
[2] Massachusetts Eye and Ear, Harvard Medical School, Boston, MA, United States
* Correspondence: nazlee_zebardast@meei.harvard.edu

Table S1. Criteria used in our package to evaluate glaucoma defects. HAP2=Hodapp-Anderson-Parrish 2; UKGTS=United Kingdom Glaucoma Treatment Study; LoGTS=Low-pressure Glaucoma Treatment Study. PDP= Pattern deviation plot, MD= Mean Deviation, TDP=Total deviation plot, TD= Total deviation, GHT = Glaucoma Hemifield Test.

**Criteria**		**VF Input Type**	**Condition for a positive outcome (Glaucomatous defect)<br>(if none of conditions met, classify as non-GL)**
1- HAP2 [1,2]	Part1	Pattern	#PDP (<0.05)>= 3 pts<br>AND<br>#PDP (<0.01) => 1 pt
	Part 2	Pattern	1- Early defect: MD= -0.01 to -6 AND<br>#PDP (<0.05) = 1 to 12 pts AND<br>#PDP (<0.01) = 1 to 4 pts<br>2- Moderate defect: MD= -6.01 to -12 AND<br>#PDP (<0.05) = 13 to 26 pts AND<br>#PDP (<0.01) = 5 to 13 pts<br>3- Severe defect: MD< -12 AND<br>#PDP (<0.05)>= 27 pts AND<br>#PDP (<0.01)>= 14pts
2- UKGTS [3,4]		Total	Sensitivity reduction at 2 or more contiguous points with TDP < 0.01
3- LoGTS [5]		Total	Cluster of 2 or more adjacent points in the TD plot at < −10 dB
4- Kang [6]		Total	Cluster of 3 or more adjacent points in the TD plot at < -5 dB
5- Foster [7]		Pattern	GHT: Outside normal limits<br>And<br>At least a cluster of 3 pts in PDP < 5%

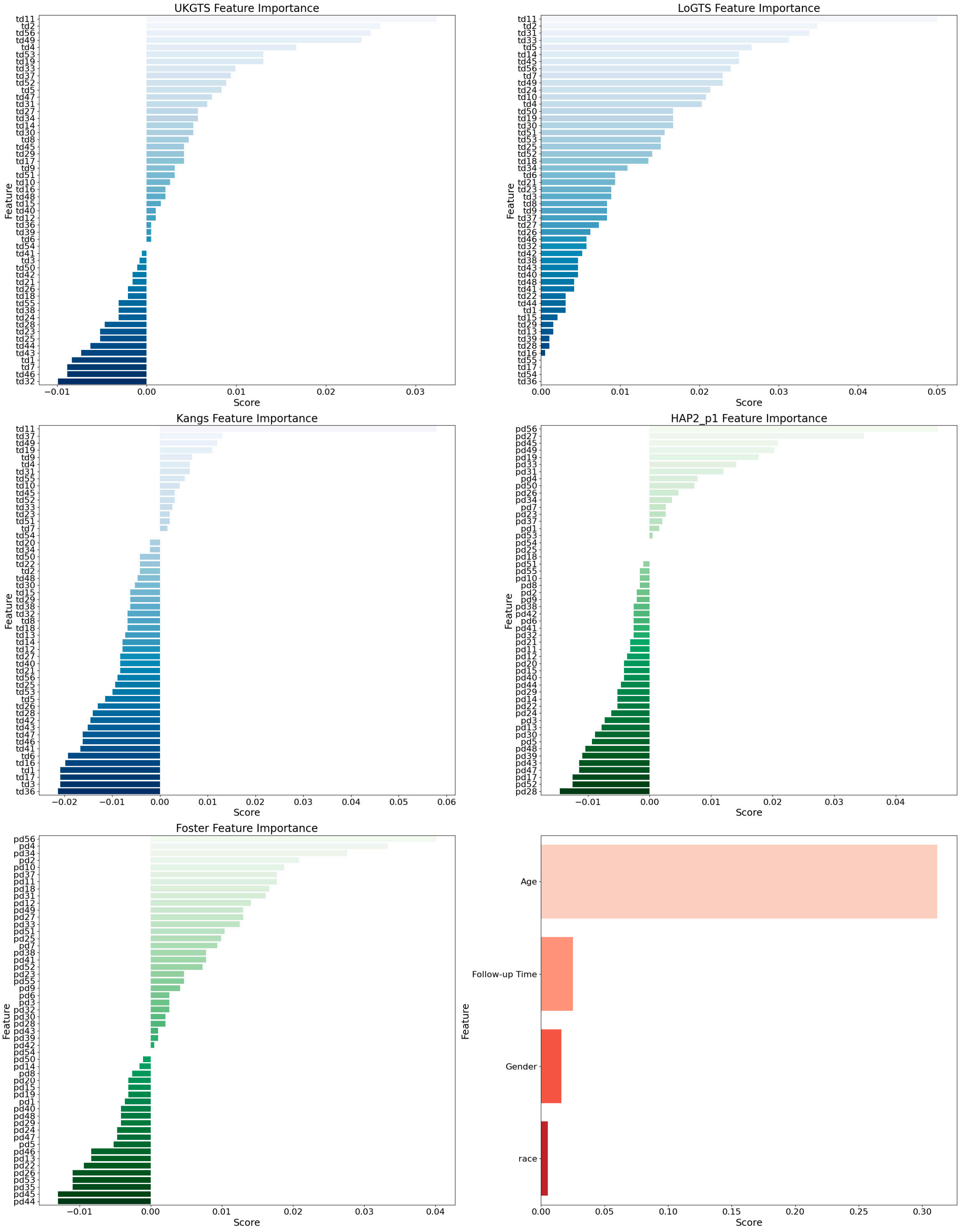

Figure S1. List of all important features extracted by MLP and their contribution to each stand-alone model.

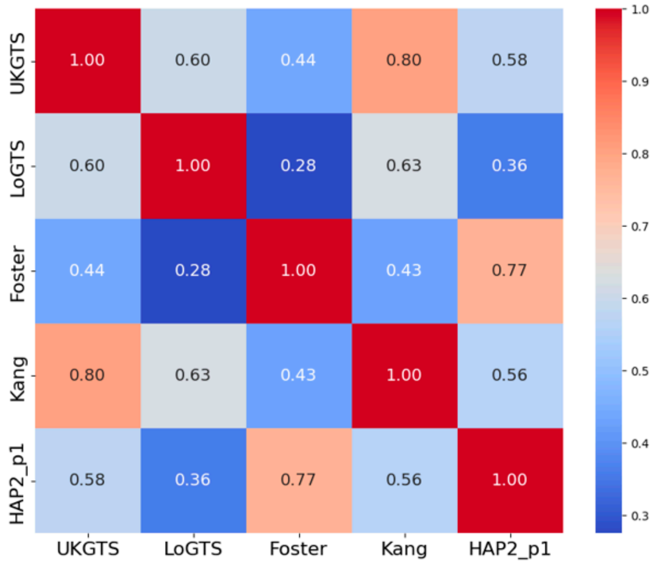

Figure S2. Correlation of predictions in each stand-alone model. Values > 0.5 indicate moderate to high correlation. Pearson correlation coefficient was used to calculate all values.

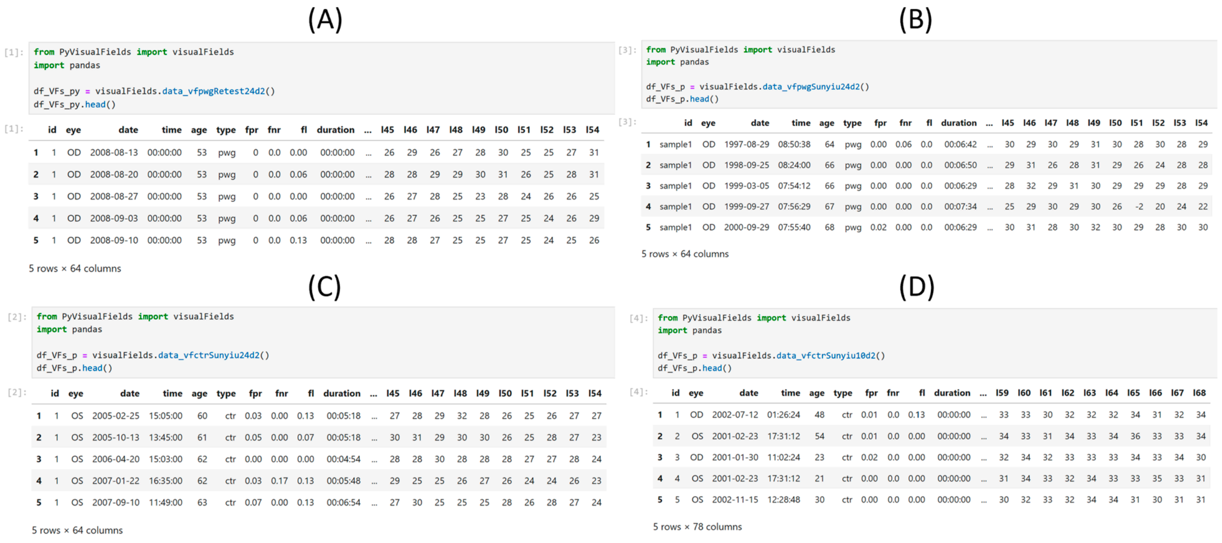

(A)

```
[1]: from PyVisualFields import visualFields
     import pandas

     df_VFs_py = visualFields.data_vfpwgRetest24d2()
     df_VFs_py.head()
```

[1]:

	id	eye	date	time	age	type	fpr	fnr	fl	duration	...	l45	l46	l47	l48	l49	l50	l51	l52	l53	l54
1	1	OD	2008-08-13	00:00:00	53	pwg	0	0.0	0.00	00:00:00	...	26	29	26	27	28	30	25	25	27	31
2	1	OD	2008-08-20	00:00:00	53	pwg	0	0.0	0.06	00:00:00	...	28	28	29	29	30	31	26	25	28	31
3	1	OD	2008-08-27	00:00:00	53	pwg	0	0.0	0.00	00:00:00	...	26	27	28	25	23	28	24	26	26	25
4	1	OD	2008-09-03	00:00:00	53	pwg	0	0.0	0.06	00:00:00	...	26	27	26	25	25	27	25	24	26	29
5	1	OD	2008-09-10	00:00:00	53	pwg	0	0.0	0.13	00:00:00	...	28	28	27	25	25	27	25	24	25	26

5 rows × 64 columns

(B)

```
[3]: from PyVisualFields import visualFields
     import pandas

     df_VFs_p = visualFields.data_vfpwgSunyiu24d2()
     df_VFs_p.head()
```

[3]:

	id	eye	date	time	age	type	fpr	fnr	fl	duration	...	l45	l46	l47	l48	l49	l50	l51	l52	l53	l54
1	sample1	OD	1997-08-29	08:50:38	64	pwg	0.00	0.06	0.0	00:06:42	...	30	29	30	29	31	30	28	30	28	29
2	sample1	OD	1998-09-25	08:24:00	66	pwg	0.00	0.00	0.0	00:06:50	...	29	31	26	28	31	29	26	24	28	28
3	sample1	OD	1999-03-05	07:54:12	66	pwg	0.00	0.00	0.0	00:06:29	...	28	32	29	31	30	29	29	29	28	29
4	sample1	OD	1999-09-27	07:56:29	67	pwg	0.00	0.00	0.0	00:07:34	...	25	29	30	29	30	26	-2	20	24	22
5	sample1	OD	2000-09-29	07:55:40	68	pwg	0.02	0.00	0.0	00:06:29	...	30	31	28	30	32	30	29	28	30	30

5 rows × 64 columns

(C)

```
[2]: from PyVisualFields import visualFields
     import pandas

     df_VFs_p = visualFields.data_vfctrSunyiu24d2()
     df_VFs_p.head()
```

[2]:

	id	eye	date	time	age	type	fpr	fnr	fl	duration	...	l45	l46	l47	l48	l49	l50	l51	l52	l53	l54
1	1	OS	2005-02-25	15:05:00	60	ctr	0.03	0.00	0.13	00:05:18	...	27	28	29	32	28	26	25	26	27	27
2	1	OS	2005-10-13	13:45:00	61	ctr	0.05	0.00	0.07	00:05:18	...	30	31	29	30	30	26	25	28	27	23
3	1	OS	2006-04-20	15:03:00	62	ctr	0.00	0.00	0.00	00:04:54	...	28	28	30	28	28	28	27	27	28	24
4	1	OS	2007-01-22	16:35:00	62	ctr	0.03	0.17	0.13	00:05:48	...	29	25	25	26	27	26	24	24	26	23
5	1	OS	2007-09-10	11:49:00	63	ctr	0.07	0.00	0.13	00:06:54	...	27	30	25	25	25	28	26	28	27	24

5 rows × 64 columns

(D)

```
[4]: from PyVisualFields import visualFields
     import pandas

     df_VFs_p = visualFields.data_vfctrSunyiu10d2()
     df_VFs_p.head()
```

[4]:

	id	eye	date	time	age	type	fpr	fnr	fl	duration	...	l59	l60	l61	l62	l63	l64	l65	l66	l67	l68
1	1	OD	2002-07-12	01:26:24	48	ctr	0.01	0.0	0.13	00:00:00	...	33	33	30	32	32	32	34	31	32	34
2	2	OS	2001-02-23	17:31:12	54	ctr	0.01	0.0	0.00	00:00:00	...	34	33	31	34	33	34	36	33	33	34
3	3	OD	2001-01-30	11:02:24	23	ctr	0.02	0.0	0.00	00:00:00	...	32	34	32	33	33	33	34	33	34	30
4	4	OS	2001-02-23	17:31:12	21	ctr	0.00	0.0	0.00	00:00:00	...	31	34	33	32	34	33	33	35	33	31
5	5	OS	2002-11-15	12:28:48	30	ctr	0.00	0.0	0.00	00:00:00	...	30	32	33	32	34	34	31	30	31	31

5 rows × 78 columns

Figure S3. Examples of HFA data inputs compatible by the proposed model. (A) data_vfpwgRetest24d2: Short-term retest static automated perimetry data. Collected from 30 glaucoma patients at the Queen Elizabeth Health Sciences Centre in Halifax, Nova Scotia, with 12 visual field tests over 12 weekly sessions [8]. (B) data_vfpwgSunyiu24d2: 24-2 static automated perimetry data from a patient with glaucoma. This dataset contains real patient data with age modified for anonymity [9]. (C) data_vfctrSunyiu24d2: A dataset of healthy eyes for 24-2 static automated perimetry, used to generate normative values (sunyiu_24d2 and related datasets). Courtesy of William H. Swanson and Mitch W. Dul [9]. (D) data_vfctrSunyiu10d2: A dataset of healthy eyes for 10-2 static automated perimetry, also provided by William H. Swanson [9].

References

1. Chang, T.C.; Ramulu, P.; Hodapp, E. *Clinical Decisions in Glaucoma*; Bascom Palmer Eye Institute Miami (FL): 2016.
2. Perkins, T.W. Clinical Decisions in Glaucoma. *Archives of Ophthalmology* **1994**, *112*, 1518-1519.
3. Garway-Heath, D.F.; Crabb, D.P.; Bunce, C.; Lascaratos, G.; Amalfitano, F.; Anand, N.; Azuara-Blanco, A.; Bourne, R.R.; Broadway, D.C.; Cunliffe, I.A. Latanoprost for Open-Angle Glaucoma (Ukgts): A Randomised, Multicentre, Placebo-Controlled Trial. *The Lancet* **2015**, *385*, 1295-1304.
4. Garway-Heath, D.F.; Lascaratos, G.; Bunce, C.; Crabb, D.P.; Russell, R.A.; Shah, A.; Investigators, U.K.G.T.S. The United Kingdom Glaucoma Treatment Study: A Multicenter, Randomized, Placebo-Controlled Clinical Trial: Design and Methodology. *Ophthalmology* **2013**, *120*, 68-76.
5. Krupin, T.; Liebmann, J.M.; Greenfield, D.S.; Rosenberg, L.F.; Ritch, R.; Yang, J.W.; Group, L.-P.G.S. The Low-Pressure Glaucoma Treatment Study (Logts): Study Design and Baseline Characteristics of Enrolled Patients. *Ophthalmology* **2005**, *112*, 376-385.
6. Kang, J.H.; Loomis, S.J.; Rosner, B.A.; Wiggs, J.L.; Pasquale, L.R. Comparison of Risk Factor Profiles for Primary Open-Angle Glaucoma Subtypes Defined by Pattern of Visual Field Loss: A Prospective Study. *Investigative Ophthalmology & Visual Science* **2015**, *56*, 2439-2448.
7. Foster, P.J.; Buhrmann, R.; Quigley, H.A.; Johnson, G.J. The Definition and Classification of Glaucoma in Prevalence Surveys. *British journal of ophthalmology* **2002**, *86*, 238-242.
8. Artes, P.H.; O'Leary, N.; Nicolela, M.T.; Chauhan, B.C.; Crabb, D.P. Visual Field Progression in Glaucoma: What Is the Specificity of the Guided Progression Analysis? *Ophthalmology* **2014**, *121*, 2023-2027.
9. Wyatt, H.J.; Dul, M.W.; Swanson, W.H. Variability of Visual Field Measurements Is Correlated with the Gradient of Visual Sensitivity. *Vision research* **2007**, *47*, 925-936.
